# A biofunctionalizable ink platform composed of catechol-modified chitosan and reduced graphene oxide/platinum nanocomposite

**DOI:** 10.3762/bjnano.8.151

**Published:** 2017-07-24

**Authors:** Peter Sobolewski, Agata Goszczyńska, Małgorzata Aleksandrzak, Karolina Urbaś, Joanna Derkowska, Agnieszka Bartoszewska, Jacek Podolski, Ewa Mijowska, Mirosława El Fray

**Affiliations:** 1 Division of Biomaterials and Microbiological Technologies, Faculty of Chemical Technology and Engineering, West Pomeranian University of Technology, Szczecin, 45 Piastów Ave., 70-311 Szczecin, Poland; 2 Nanomaterials Physicochemistry Department, Faculty of Chemical Technology and Engineering, West Pomeranian University of Technology, Szczecin, 45 Piastów Ave., 70-311 Szczecin, Poland; 3 NZOZ “Meditest. Diagnostyka Medyczna”, Bronisławy 14 D, 71-533, Szczecin, Poland

**Keywords:** biosensing, catechol, chitosan, graphene, piezoelectric printing

## Abstract

We present an ink platform for a printable polymer–graphene nanocomposite that is intended for the development of modular biosensors. The ink consists of catechol-modified chitosan and reduced graphene oxide decorated with platinum nanoparticles (rGO–Pt). We modified the chitosan with catechol groups, in order to obtain adhesive properties and improve solubility. Dispersions of rGO–Pt in ethylene glycol were admixed with an aqueous solution of modified chitosan to yield an ink that is suitable for non-contact piezoelectric printing using a commercial microplotter (Sonoplot GIX Microplotter Desktop). As a proof of concept, printed patterns were biofunctionalized with DNA oligonucleotide probes for *Streptococcus agalactiae* (Group B streptococcus) using glutaraldehyde as a linker. Confocal microscopy revealed the successful hybridization of complementary polymerase chain reaction (PCR) products and low non-specific binding. Our results demonstrate that catechol-modified chitosan/rGO–Pt nanocomposites can be used as inks for piezoelectric printing and facilitate the attachment of biorecognition elements for biosensor applications.

## Introduction

Biosensors are a key enabling technology for the paradigm shift towards decentralized, personalized and targeted medicine. They offer the potential to utilize the wealth of information provided by modern molecular biology (genomics and proteomics, in particular) during the crucial process of diagnosis. Importantly, an ideal biosensor platform needs to not only be sensitive and specific, but also flexible and affordable [1].

The past ten years have seen the growth of several technologies that hold much promise for the field of biosensors. The seminal work of Novoselov and Geim [2] introduced graphene, a two-dimensional sheet form of carbon. From the point of view of biosensing, graphene possesses a number of extremely attractive properties [3], including large specific surface area and high electron mobility. Importantly, graphene can be incorporated into polymer–graphene nanocomposites [4], gaining the additional properties of the polymer matrix, in addition to easing handling and reducing cost. Equally important have been advances in bioprinting [5], such as micro-contact printing, laser direct writing, and inkjet printing, providing cheaper, rapid alternatives to traditional lithography techniques. Particularly appealing are piezoelectric approaches, including inkjet [6] and microplotter systems [7], as they offer non-contact deposition under mild conditions. Importantly, these techniques are versatile, allowing for a broad range of inks, and scalable, as well as cost effective, thanks to reduced material waste and no mask or tooling requirement.

Here we present a chitosan–catechol/graphene nanocomposite suitable for use as ink for piezoelectric non-contact printing that can serve as a platform for biosensor development. First, we prepare dispersions of reduced graphene oxide (rGO) decorated with platinum nanoparticles (rGO–Pt) in ethylene glycol (EG). As the polymer matrix, we utilize chitosan (CHI), a polycationic biopolymer that provides excellent film-forming properties and easy-to-functionalize amine groups [8]. However, we first chemically modify the chitosan to add catechol pendant groups to the chitosan polymer chains [9], in order to improve water solubility, as well as provide adhesive properties to a variety of substrates [10]. The ink is formed by admixing rGO–Pt dispersions in ethylene glycol with the aqueous polymer solution and can be printed by using a commercial non-contact piezoelectric microplotter. While the application of chitosan/graphene nanocomposites for biosensing is established [4], to our knowledge, this is the first time that such a nanocomposite has been formulated as ink. Importantly, the printed nanocomposite has ample functional groups to chemically conjugate various biorecognition elements and resists the wash steps inherent in biosensing applications. As a proof of concept, we biofunctionalize printed patterns with DNA oligonucleotide probes for *Streptococcus agalactiae* (Group B streptococcus, GBS) and visualize the hybridization of fluorescently-labeled complementary polymerase chain reaction (PCR) amplicons using confocal microscopy.

## Results and Discussion

### rGO–Pt Dispersions

Our ink system is composed of two components: the rGO–Pt dispersion and the polymer solution. While graphene oxide can be readily dispersed in aqueous solutions, graphene and rGO require appropriate organic solvents [11]. *N*-methyl-2-pyrrolidone (NMP) is perhaps the ideal solvent for the exfoliation of graphite and graphene. However, the aggressive nature of this solvent led us to choose ethylene glycol (EG), as it performed best in our initial dispersion tests, in comparison to isopropyl alcohol and dimethylformamide (data not shown). We prepared our dispersions following a modified version of the NMP graphene dispersion protocol used by Torrisi et al. [12], consisting of alternating bath sonication and centrifugation. We were able to obtain rGO–Pt dispersions in EG and, using UV–vis spectrophotometry, estimated the dispersion concentration to range from 0.08 to 0.45 g/L (Table 1).

**Table 1:** Initial amount of rGO–Pt in EG and amount obtained in dispersion as estimated from absorbance at 660 nm.

initial amount of rGO–Pt in EG	dispersion concentration

2 g/L	0.08 g/L
5 g/L	0.13 g/L
10 g/L	0.45 g/L

**Figure 1:**
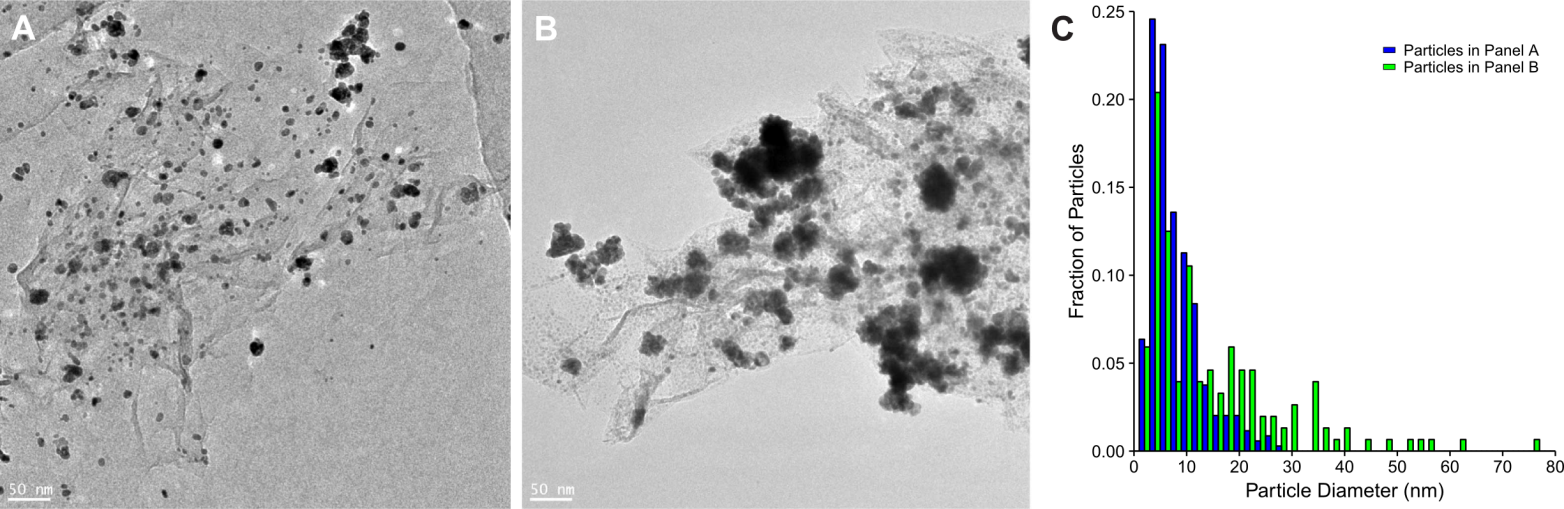
Representative TEM micrographs of: A) final rGO–Pt dispersion, B) sediment rGO–Pt. The scale bars represent 50 nm. C) Histogram of Pt particles observed in panel A (blue) and panel B (green) (bin width = 2).

In order to verify that the sonication and/or centrifugation process did not adversely affect the integrity of rGO–Pt, particularly the Pt nanoparticles, we examined the dispersed material and sediment after centrifugation using transmission electron microscopy (TEM) (Figure 1). We confirmed the presence of Pt nanoparticles within the dispersed material (Figure 1A), with individual particles having a mean diameter of 6.0 ± 2 nm, though some aggregates are also visible. The dispersed material has a more homogenous distribution of Pt nanoparticles, as compared to the sediment material (Figure 1B), where large aggregates of Pt nanoparticles are visible (see Figure 1C for a histogram of the particles in panels A and B). It appears that the sonication and centrifugation process may serve as a form of selection for more homogenously decorated rGO.

### Catechol-modified chitosan

As the polymer component of our ink system we chose chitosan for its excellent film forming properties and abundant amine groups [8]. Additionally, we modified the chitosan with catechol groups, in order to gain the additional adhesive properties of these groups and improve water solubility [9]. Following the methodology of Kim et al. [9], we used carbodiimide chemistry to couple hydrocaffeic acid (HCA) to the amine groups of chitosan. Based on this prior work, our goal was to obtain degree of conjugation (DoC) of approximately 7–13%, because a higher DoC reduces solubility and results in gelation. The DoC is defined as the amount (in mol %) of chitosan repeating units carrying a catechol group, as calculated from NMR data (see Supporting Information File 1). The obtained ^1^H NMR spectrum (see Figure S3, Supporting Information File 1) confirms the reaction product and we calculate the DoC as 13%. Despite being on the high end of our desired range, the obtained catechol-modified chitosan (CHI-HCA) readily dissolved in water, up to 3% w/v, which is comparable to the results reported by Kim and co-workers [9].

### Ink preparation and printing

**Figure 2:**
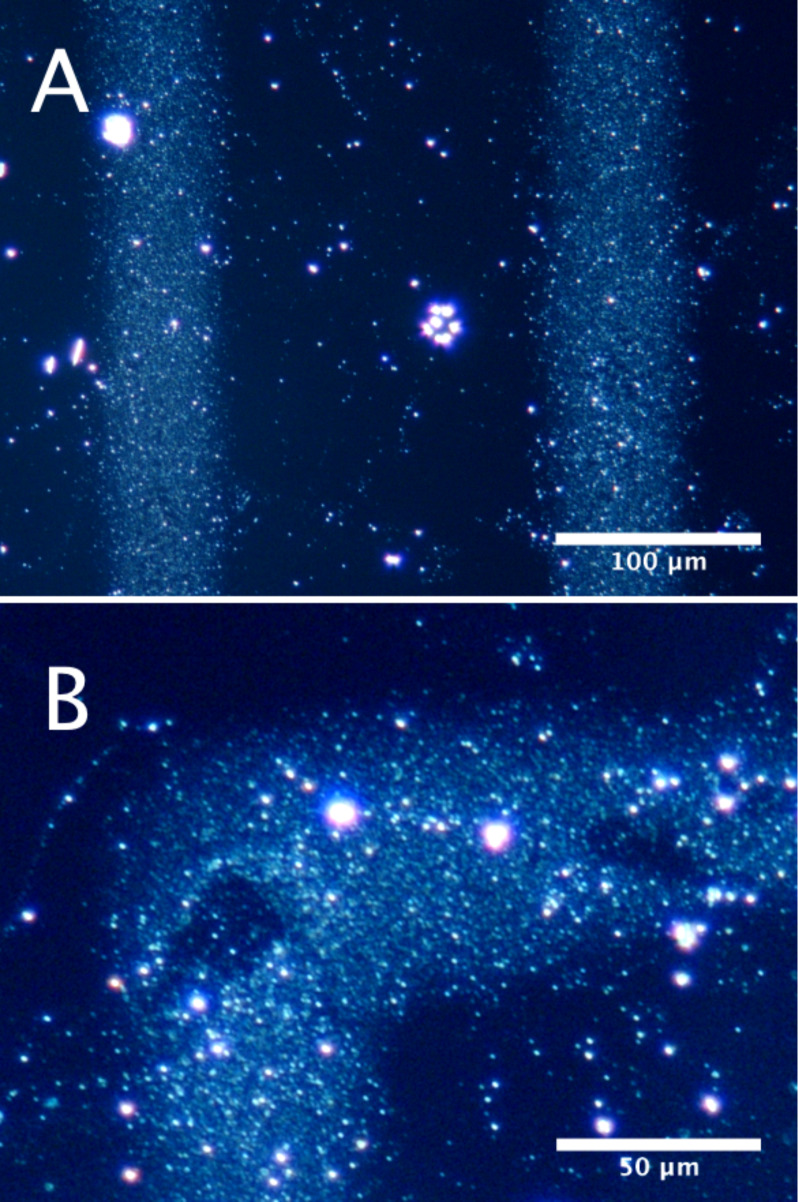
A) Representative dark-field image of pattern printed using rGO–Pt ink. B) Enlarged detail of the “elbow” portion of a printed pattern, showing some defects. Bright white spots are imperfections in the glass and/or dust.

Inks were prepared by admixing the rGO–Pt dispersions with a 2% w/v aqueous CHI-HCA solution in a 3:1 ratio, followed by an additional 30 min bath sonication. This binary solvent mixture of EG and water, each possessing differing boiling points and surface tensions, was intended to reduce the formation of so-called “coffee rings,” which are common when printing dispersions [13]. Inks were readily taken up by the 60 μm glass dispensers of the Sonoplot GIX Microplotter Desktop, a commercial piezoelectric microplotter. All further presented results were obtained using ink prepared with the rGO–Pt dispersion of highest concentration (0.45 g/L). Printing tests were conducted using pristine glass microscope slides as substrates. We noted excellent fluid bridge formation between the dispenser and the glass substrate, a crucial step in ensuring successful non-contact printing using our microplotter. Following printing, samples were thermally annealed at 170 °C for 5 min, again with the goal of minimizing “coffee ring” formation [12]. Following annealing, the printed structures were invisible to the naked eye or under bright-field microscopy. As a result, in order to examine the printed structures and check for possible “coffee ring” formation, we utilized dark-field microscopy. We observed homogenous stripes, consistent with the design and tip dimensions. Figure 2 shows a representative dark-field image for the printed and annealed ink. Subtle defects in the printed structure can be observed at the “elbows” of the print (Figure 2B). However our images are broadly similar to those reported by Torrisi et al. [12] for inkjet-printed graphene ink on Si/SiO_2_ and confirm the lack of “coffee ring” effects.

We developed our ink for a commercial microplotter system (Sonoplot GIX Microplotter Desktop), which uses a piezoelectric crystal to pump ink out of a glass micropipette [7]. This system does not generate droplets like an inkjet printer, instead it deposits continuous features via the fluid bridge formed between the substrate and dispenser tip. This allows for a broad range of viscosity of inks (up to 0.450 Pa·s), without concern for filaments/ligaments or satellite droplets. However, in order to assess the suitability of our ink for the more commonly used piezoelectric inkjet printing, we can estimate the *Z* parameter (the reciprocal of the Ohnesorge number (Oh), the ratio of the Reynolds number (Re) to the square root of the Webber number (We)), which predicts stable drop formation [14]:

[1]
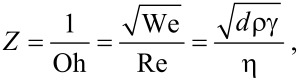


where *d* is the nozzle diameter and ρ, γ, and η represent the density, surface tension, and viscosity of the ink, respectively. We were not able to measure directly the viscosity and surface tension of our ink as formulated. However, we were able to estimate the values of the parameters based on the values provided by Jang et al. for EG/water mixtures [14] and the viscosity of the chitosan polymer solution (0.049 Pa·s). For a typical nozzle diameter range of 20 to 100 μm, we estimate the *Z* parameter for our ink as formulated, to be in the range of 1.3 to 2.9. In general, *Z* in the range of 1 to 10 is commonly considered to yield inkjet-printable ink [6]. However, Jang et al. describe the drop behavior of an EG/water ink system over a wider range of *Z* values [14]. For *Z* values below 4, they observe that single drops are generated, but with long ligaments, thus requiring larger minimum standoff distances, resulting in greater error. Thus, while it should be possible to use our ink for inkjet printing, it may be necessary to reduce the viscosity, in order to increase the *Z* value and reduce ligament length, for example by reducing the concentration of CHI-HCA polymer.

### Ink biofunctionalization and confocal imaging

**Figure 3:**
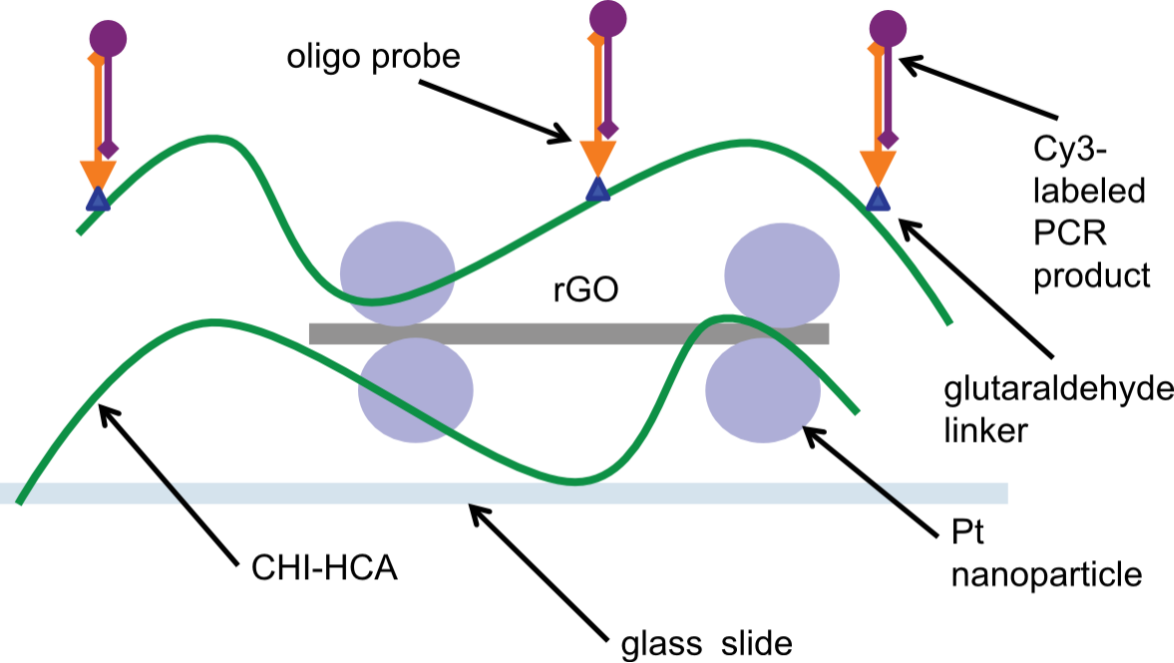
Schematic of printed catechol-modified chitosan/rGO–Pt nanocomposite, functionalized with DNA oligonucleotide probes and hybridized with Cy3-labeled complementary PCR product.

The biorecognition interface of any biosensor plays a crucial role, as it is the feature that enables the selective detection of the target biomolecules. Our ink platform is designed to be modular; various possible biorecognition elements can be conjugated to the remaining amine groups of the catechol-modified chitosan, such as enzymes, antibodies, DNA probes, or aptamers. As a proof of concept, we reacted our amine-terminated DNA oligonucleotide probes for Group B streptococcus (GBS) to the printed, annealed nanocomposite ink stripes using glutaraldehyde as a linker [15]. Next, the modified structures were allowed to hybridize with Cy3-fluorescence-labeled, PCR-amplified complementary DNA extracted from GBS positive (GBS+) clinical isolates or non-complementary negative control (see Figure 3 for the scheme).

Our goal was to confirm that it was possible to conjugate biorecognition molecules to the ink and that they would remain functional. Additionally, we wanted to test whether the catechol-modified chitosan matrix would foster non-specific binding of the target analyte, in this case DNA. We chose a DNA detection system, since this is likely the worst-case scenario from the point of view of non-specific binding; chitosan is known to interact with and bind DNA, leading to its use in non-viral gene delivery [16].

**Figure 4:**
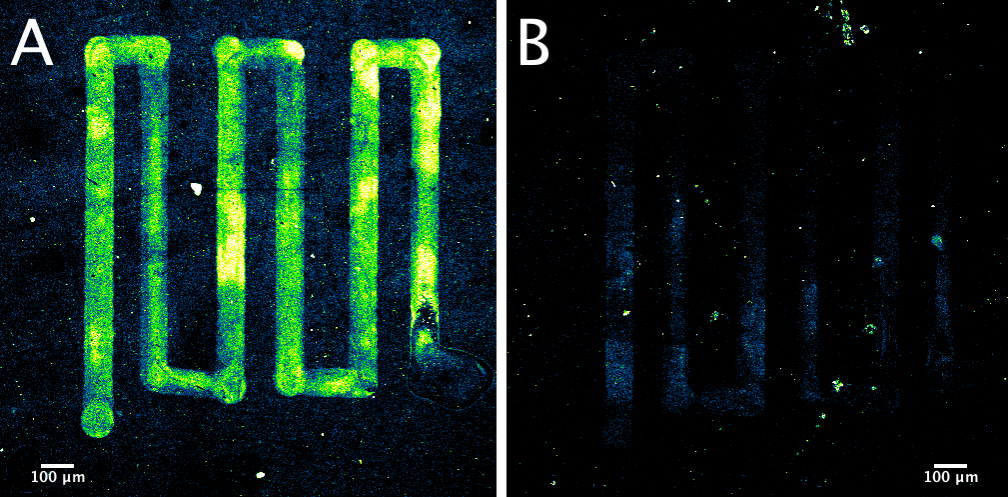
Confocal microscopy of printed, annealed rGO–Pt ink functionalized with DNA oligonucleotide probes. A) Results of hybridization with complementary, GBS+ Cy3-labeled PCR product. B) Results of hybridization with non-complementary, control Cy3-labeled PCR product.

As this is a proof-of-concept study, we utilized confocal microscopy in order to assess the hybridization of Cy3 fluorescence-labeled complementary PCR product (Figure 4). This method, while not practical for real-world biosensing applications, allowed us to visualize uniformity and distribution of the signal that a fluorometer or an electrochemical measurement would not provide. Further, we wanted to verify the adhesive properties of the ink provided by the catechol-modified chitosan by visually confirming that the printed structures endure the process, including all wash steps. These properties are crucial in enabling possible applications in microfluidic chips, another key biosensor enabling technology [17].

In the case of hybridization with complementary, GBS+ PCR product, we observed a strong signal from the printed pattern (Figure 4A, background-subtracted mean ± SD of three ROIs: 109 ± 6 arb. unit), while hybridization with non-complementary, control PCR product (Figure 4B) resulted in a markedly weaker signal (background subtracted mean ± SD of three ROIs: 11 ± 6 arb. unit).

As a result, we conclude that our ink platform was successfully modified to add a biorecognition interface, namely the GBS oligonucleotide probes, and that this interface remained functional, as evidenced by strong signal from hybridized Cy3-labeled complementary PCR product. We confirmed the adhesive properties granted by the catechol modification of the chitosan matrix. Printed patterns utilizing ink prepared with thiol-modified chitosan instead or no chitosan, just rGO–Pt dispersed in EG, were washed off during the course of the functionalization/hybridization process.

Importantly, we observed no strong non-specific binding between the non-complementary PCR product and the catechol-modified chitosan matrix (Figure 4B). Chitosan/DNA interactions are largely driven by electrostatic interaction and, as a result, solution pH value, degree of deacetylation and molecular weight all play important roles [18]. Following biofunctionalization via glutaraldehyde linker to the amine groups of chitosan, it is likely that few amine groups remain free, as glutaraldehyde was in excess, and of those remaining, less than half will be protonated during the hybridization, as the buffer pH value of 7 is greater than the p*K*_a_ of chitosan (p*K*_a_ 6–6.5). Further, our choice of chitosan with very low molecular weight (5 kDa) may also contribute, as the observed binding constant for chitosan and DNA depends on the molecular weight of chitosan [18].

## Conclusion

We present a novel polymer–graphene nanocomposite ink platform for modular biosensor development. The ink consists of rGO decorated with Pt nanoparticles dispersed in ethylene glycol, admixed with an aqueous solution of catechol-modified chitosan. Using our commercial piezoelectric microplotter, the ink formed an excellent fluid bridge and was readily printable. Following thermal annealing, the resultant chitosan/graphene nanocomposite did not develop “coffee rings.” Based on the estimated *Z* parameter of our ink, we expect that it will yield single drops and, as a result, can also be utilized in inkjet printers with only minor tuning. We demonstrate that the printed ink can be functionalized with biorecognition elements and, importantly, that the printed structures resist washing following biofunctionalization and hybridization, thanks to the adhesive properties of the catechol moiety. Our proof of concept involving DNA oligonucleotide probes demonstrates that the printed structures can be functionalized in a way that preserves probe function and that the ink itself does not yield substantial non-specific binding of DNA. Further studies will be conducted to evaluate the potential of this ink platform for other biosensing systems, including electrochemical detection and/or glucose biosensing.

## Experimental

### Preparation of reduced graphene oxide (rGO) functionalized with Pt nanoparticles (rGO–Pt)

**Figure 5:**
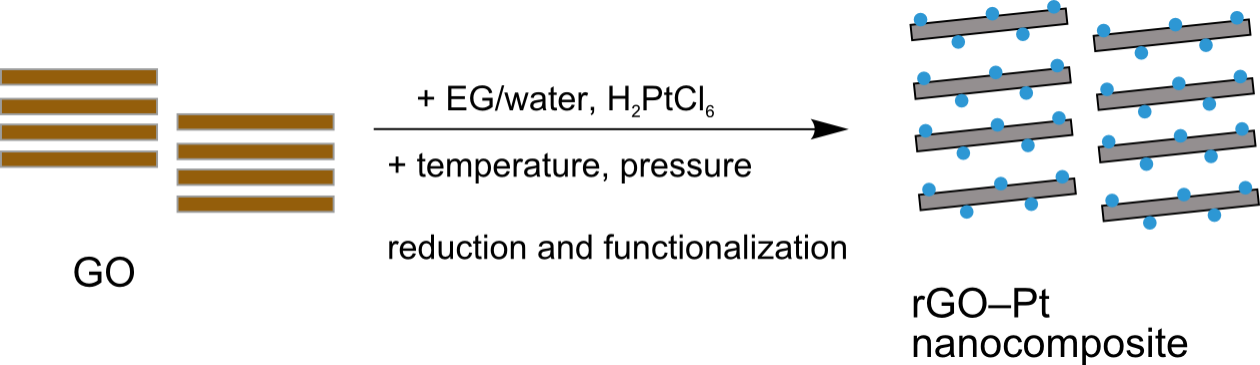
Simultaneous reduction and functionalization of GO to rGO–Pt nanocomposite.

Graphene oxide (GO) was synthesized by oxidation of graphite flakes (Aesar, 325 mesh), using a modified Hummers method [19], as described previously [20]. Next, GO was reduced and functionalized with Pt nanoparticles (Figure 5). First, 75 mg GO was dispersed in a mixture of ethylene glycol (EG) and water (50 mL of EG, 100 mL of H_2_O) and bath-sonicated for 2 h to achieve homogeneous dispersion. Simultaneously, 75 mg H_2_PtCl_6_ was dispersed in 150 mL EG/water solution (1:2 volume ratio) and poured into the homogeneous suspension of GO (150 mL). After sonication for 30 min, the mixture was poured into an autoclave and stirred for 24 h at 110 °C. The obtained material was separated by filtration, followed by washing with water and ethanol and drying in air at 100 °C. See Supporting Information File 1 for rGO–Pt characterization.

### rGO-Pt Dispersions

Dispersions were prepared based on the work of Torrisi et al. [12], but in ethylene glycol. Briefly, 2 g/L, 5 g/L, and 10 g/L of rGO–Pt was dispersed in EG via bath sonication (8 h, Polsonic Sonic-10, 40 kHz), followed by centrifugation (1 h, MPW 223e, 2500 RCF), another round of sonication (1 h), and finally another round of centrifugation. Dispersion concentrations were assessed via UV–vis spectrophotometry [21]. Transmission electron microscopy (TEM) (FEI Tecnai F30, Frequency Electronics Inc.) was employed to examine the morphology of the samples and confirm that the dispersion process did not remove Pt nanoparticles from rGO–Pt. The obtained images were analyzed using Fiji [22] following the NIST guidelines [23]:

1. noise was reduced by processing with “Despeckle,” followed by five rounds of “Smooth,”
2. automated thresholding was performed using the Otsu algorithm to obtain binary data,
3. binary data was further processed with “Fill holes” and “Watershed”, and
4. particle analysis was carried out using “Analyze particles” (Size: 4 nm^2^ to infinity, circularity: 0.5 to 1, exclude on edges).

Three higher magnification fields of view (260 nm × 260 nm, 122 particles) were first analyzed to determine the size of individual particles, with 4.6 nm^2^ being the minimum size recorded, providing the needed minimum size setting (4 nm^2^) for remaining analysis. Likewise, the high measured circularity (0.84) led us to convert measured particle areas to particle diameters.

### Preparation of catechol-modified chitosan (CHI-HCA)

Catechol modification of chitosan (chitosan oligosaccharide lactate, 5 kDa, 90% degree of deacetylation, Sigma) was carried out by using *N*-(3-dimethylaminopropyl)-*N*′-ethylcarbodiimide (EDC) coupling of hydrocaffeic acid (HCA), based on the work of Kim and co-workers [9]. Briefly, the conjugation reaction was carried out using a 2:1:1 molar ratio of chitosan amine functional groups to HCA and EDC, in an aqueous solution (final pH 4.5, 25% ethanol), at room temperature for ca. 18 h. The product was purified via dialysis (three changes over 24 h, 3.5 kDa membrane, SpectraPor) and lyophilized. The degree of conjugation (DoC, defined as the mol % of chitosan repeating units carrying a catechol group) was calculated from ^1^H NMR data (see Supporting Information File 1 for details).

### Preparation of ink and printing

The ink was prepared by admixing the rGO–Pt dispersion with a 2% w/v aqueous CHI-HCA solution in a 3:1 v/v ratio, followed by additional 30 min of bath sonication. Sonoplot GIX Microplotter Desktop (60 μm dispenser, at a resonant frequency determined by the instrument of ca. 440 kHz, 2 V_pp_) was used to print ink on glass slides (pristine, Comex). Based on the work of Torrisi et al. with NMP [12], in order to avoid “coffee ring” formation, EG, which has a similar boiling point as NMP, was removed by thermal annealing in an oven at 170 °C for 5 min. Printed patterns were imaged using dark field microscopy (Nikon MM-40).

### Biofunctionalization of printed features

To demonstrate the potential of our ink platform, we conjugated printed patterns with DNA oligonucleotide probes and visualized the hybridization of the fluorescently labeled PCR product, following the scheme illustrated in Figure 3. See Supporting Information File 1 for description of probe design, as well as DNA isolation and amplification. We used a similar approach as Singh et al. [15], who biofunctionalized a spin-coated chitosan/graphene oxide nanocomposite intended for electrochemical biosensing. Briefly, the printed structures were modified with glutaraldehyde (2% aqueous solution, 2 h, followed by three washes with distilled water) to react with amine groups of chitosan and serve as a linker. Next, the structures were incubated for 1 h with a 30 μM solution of amine-terminated oligonucleotide probes for a region of the *S. agalactiae cfb* gene (Group B streptococcus (GBS)), followed by three washes in distilled water. Finally, the Cy3-labelled PCR product (amplified complementary DNA extracted from GBS positive (GBS+) clinical isolates or non-complementary negative control) in hybridization buffer (5× saline-sodium citrate buffer (SSC), 0.1% SDS, 10% formamide) was incubated with functionalized ink structures for 1 h, followed by three washes with wash buffer (1X SSC, 0.1% SDS). The slides were allowed to dry in air and were imaged using Leica TCS SP8 confocal microscope. The obtained 8-bit images were analyzed using Fiji.

## Supporting Information

Supporting Information contains details of the rGO–Pt nanocomposite characterization, NMR analysis of CHI-HCA, GBS probe design and DNA isolation/amplification.

File 1 Additional experimental data.
