# Supplementary material for: A biofunctionalizable ink platform composed of catechol-modified chitosan and reduced graphene oxide/platinum nanocomposite
Source: Beilstein J Nanotechnol. 2017 Jul 24;8:1508–14. doi: 10.3762/bjnano.8.151 (PMC5550807; doi:10.3762/bjnano.8.151)
Supplement: File 1 — Additional experimental data. [file Beilstein_J_Nanotechnol-08-1508-s001.pdf]

Supporting Information

for

A biofunctionalizable ink platform composed of catechol-modified chitosan and reduced graphene oxide/platinum nanocomposite

Peter Sobolewski*[,1], Agata Goszczyńska[1], Małgorzata Aleksandrzak[2], Karolina Urbaś[2], Joanna Derkowska[§,3], Agnieszka Bartoszewska[3], Jacek Podolski[3], Ewa Mijowska[2] and Mirosława El Fray[1]

Address: [1]Division of Biomaterials and Microbiological Technologies, Faculty of Chemical Technology and Engineering, West Pomeranian University of Technology, Szczecin, 45 Piastów Ave., 70-311 Szczecin, Poland; [2]Nanomaterials Physicochemistry Department, Faculty of Chemical Technology and Engineering, West Pomeranian University of Technology, Szczecin, 45 Piastów Ave., 70-311 Szczecin, Poland and [3]NZOZ "Meditest. Diagnostyka Medyczna", Bronisławy 14 D, 71-533, Szczecin, Poland

Email: Peter Sobolewski* - psobolewski@zut.edu.pl

* Corresponding author

[§] Present address: Clinic of Rheumatology, Internal Medicine, and Geriatrics, SPSK Nr. 1, Pomeranian Medical University, Unii Lubelskiej 1, 71-252 Szczecin, Poland

Additional experimental data

Characterization of the rGO–Pt material

Figure S1 presents Raman spectra of graphite, graphene oxide (GO), and reduced graphene oxide (rGO) functionalized with Pt nanoparticles (rGO–Pt). Three peaks are observed in the spectrum of graphite. The D band at 1311 cm^{-1}, the G band at 1577 cm^{-1} and the 2D band at 2643 cm^{-1}. The first band is a breathing mode of A_{1g} symmetry involving phonons near the K zone boundary [1]. The G mode originates from the in-plane vibration of sp^2-hybridized carbon atoms and is a doubly degenerate phonon mode (E_{2g} symmetry) at the Brillouin zone center [2]. The 2D band comes from a two-phonon double resonance Raman process [3]. After oxidation treatment the G, D and 2D bands undergo significant changes. The G band becomes broader and up-shifts, as compared to that of graphite (1600 cm^{-1}), which may be associated with isolated double bonds resonating at higher frequencies. The higher intensity of the D band in GO Raman spectrum is related with the presence of certain fraction of sp^3-hybridized carbon atoms because of amorphization of graphite during oxidation process [4]. The 2D band of graphene oxide shifts to 2613 cm^{-1} and significant decrease in its intensity is observed as a result of oxidation of graphite and formation of material with lower number of graphene layers. It is reported in the literature that after reduction of graphene oxide, the G band shifts back near to the position of the G band in graphite, which is explained as graphitic "self-healing" [5]. However, in case of the rGO–Pt nanocomposite, the G band is apparent at 1596 cm^{-1}, a result of the functionalization with Pt nanoparticles. The high intensity of the D band in the spectrum of rGO–Pt corresponds to formation of defects during thermal reduction of graphene oxide and functionalization with Pt nanoparticles. The position, intensity and shape of the 2D band did not change compared to graphene oxide, indicating that number of graphene layers did not change during functionalization process.

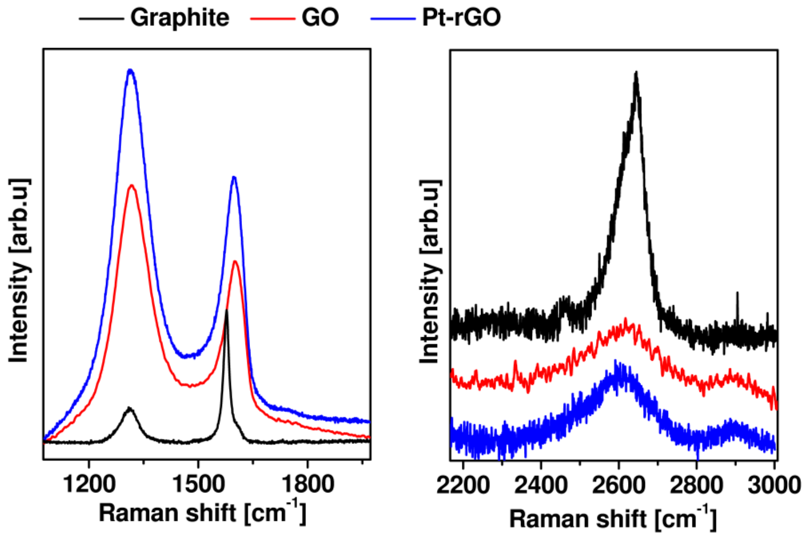

Figure S1: Raman spectra of graphite, graphene oxide (GO) and rGO–Pt nanocomposite.

Figure S2 depicts XRD patterns obtained from GO and rGO–Pt. The spectrum of GO contains the intense and sharp peak at 2θ = 11.47°, corresponding to *d*-spacing of 0.77 nm (002). The small diffraction peaks at 2θ = 26.7° and 42.7° indicate a short-range order in stacked graphene layers. The pattern for rGO–Pt shows a strong reduction of the 11.47° peak intensity, associated with the removal of oxygen-containing functional groups. In addition, few new peaks appeared at around 39.7°, 46.3°, 67.6°, and 81.5°, which can be indexed to (111), (200), (220), and (311) reflections of pure Pt with face-centered-cubic (fcc) phase [6].

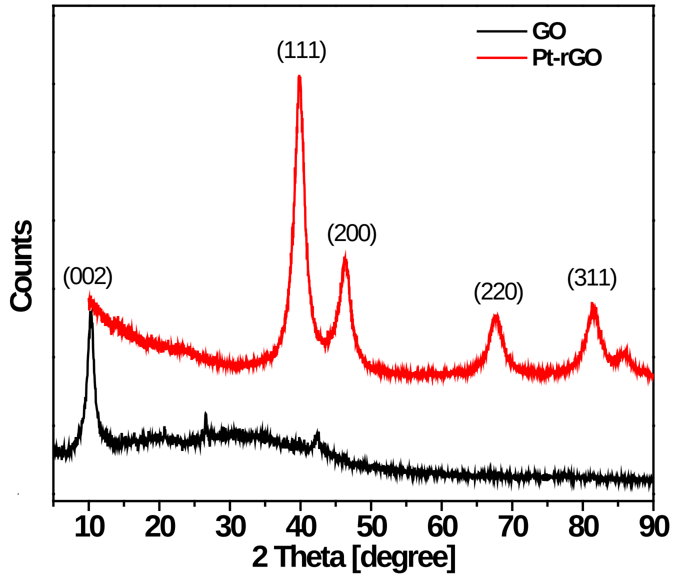

Figure S2: XRD patterns of graphene oxide (GO) and rGO-Pt nanocomposite.

NMR spectroscopy of catechol-modified chitosan

^1H NMR measurements were performed on a TM Bruker DPX 400 spectrometer, operating at 400 MHz, in deuterium oxide (D_2O) solution at a temperature of 25 °C.

^1H NMR (400 MHz, D_2O) δ 6.75–6.56 (br m, 3H, Ar), 4.05–3.33 (br m, H-3, H-4, H-5, H-6 of GlcN and H-2, H-3, H-4, H-5, H-6 GlcNAc), 3.02 (br s, 1H, H-2 of GlcN), 2.65 (t, *J* = 7.7 Hz, 2H, -C$\underline{H}_2$CH$_2$Ar), 2.48 (t, *J* = 7.6 Hz, 2H, -CH$_2$C$\underline{H}_2$Ar), 1.94 (s, 3H, CH_3 of NHAc).

The spectrum (Figure S3) shows the characteristic signal of the protons in the *N*-acetyl group (GlcNAc) at δ ≈ 1.94. The resonances of the ring protons H-3 to H-6 of GlcN and H-2 to H-6 of GlcNAc are present in the middle of the spectrum (δ = 4.05–3.33), forming a group of broad, overlapping signals. The remaining H-2 ring protons of GlcN units are shifted to lower values (δ ≈ 3.02). Finally, three hydrogens attached to an aromatic ring of the catechol group appear as a complex multiplet in the region between 6.75 ppm and 6.56 ppm. Additional signals appearing in the region of the spectrum δ = 1.33–0.95 are likely resonances of methyl groups of residual lactate from the starting chitosan oligosaccharide lactate substrate (and/or conjugates).

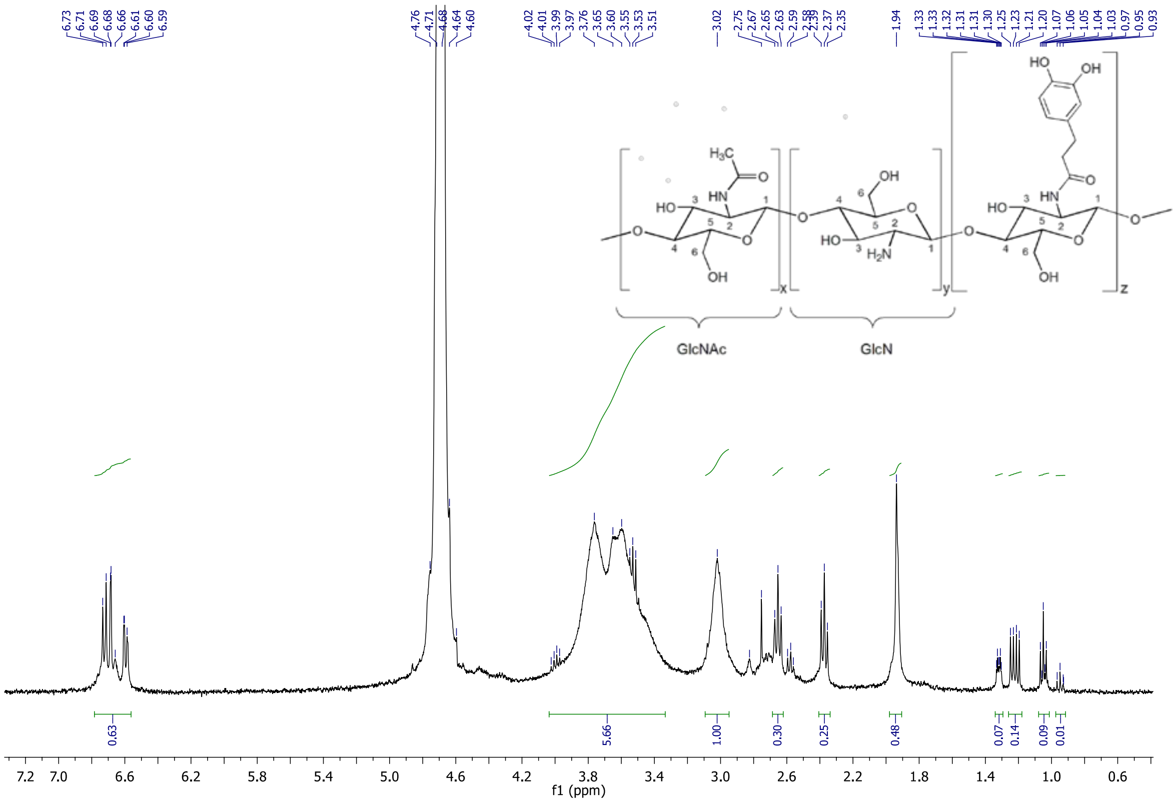

Figure S3: ^{1}H NMR spectrum of catechol-modified chitosan.

The degree of HCA conjugation, defined as the mol % of the chitosan repeating units carrying HCA was calculated from the ^{1}H NMR spectrum by comparing the relative peak area of the CS-HCA catechol group (3H, aromatic ring proton, δ 6.75–6.56 ppm) and chitosan acetyl group (3H, $NHCOOCH_3$, δ 1.94 ppm) on a chitosan backbone, taking into account the degree of deacetylation (90%).

Oligonucleotide probe design

Primer and probe sequences were designed to target a region of the *cfb* gene of *Streptococcus agalactiae*, encoding group-B streptococcal CAMP factor. Probe design and specificity were assessed using the BLASTN tool (National Center for Biotechnology Information). Oligonucleotide synthesis was performed by Genomed S. A., with 6-carbon spacer and terminal amine group (C6-NH2). Primers were also prepared for a region of human *HBE1* gene, to serve as a negative control.

DNA isolation and amplification

DNA was extracted from *S. agalactiae* positive (GBS+) clinical isolates using automated extraction platform, DNA/RNA easyMAG (Biomerieux). Amplification was conducted using RT-PCR with Cy3-labeled reverse primer for *cfb* (GBS+ samples) and FastStart Essential mix (Roche). As a negative control, RT-PCR amplification was carried out using Cy3-labeled reverse primer for *HBE1*, yielding similar amounts of amplicon. Thermal cycle conditions (95 °C – 15 min; 50 cycles, 60 °C – 1 min, 72 °C – 2 sec; 95 °C 30 sec, 40 °C) and primer concentration were chosen following manufacturer instructions.

References

1. Ferrari, A. C.; Robertson, J. *Phys. Rev. B* **2000,** *61,* 14095. doi:10.1103/PhysRevB.61.14095
2. Ni, Z.; Wang, Y.; Yu, T.; Shen, Z. *Nano Res.* **2008,** *1,* 273–291. doi:10.1007/s12274-008-8036-1
3. Narula, R.; Reich, S. *Phys. Rev. B* **2008,** *78,* 165422. doi:10.1103/PhysRevB.78.165422
4. Kudin, K. N.; Ozbas, B.; Schniepp, H. C.; Prud’homme, R. K.; Aksay, I. A.; Car, R. *Nano Lett.* **2008,** *8,* 36–41. doi:10.1021/nl071822y
5. Naebe, M.; Wang, J.; Amini, A.; Khayyam, H.; Hameed, N.; Li, L. H.; Chen, Y.; Fox, B. *Sci. Rep.* **2014,** *4,* 4375. doi:10.1038/srep04375
6. Hyde, T. *Platinum Met. Rev.* **2008,** *52,* 129–130. doi:10.1595/147106708X299547
